# Effects of *Streptococcus pneumoniae* Strain Background on Complement Resistance

**DOI:** 10.1371/journal.pone.0024581

**Published:** 2011-10-13

**Authors:** Catherine Hyams, Sophia Opel, William Hanage, Jose Yuste, Katie Bax, Birgitta Henriques-Normark, Brian G. Spratt, Jeremy S. Brown

**Affiliations:** 1 Centre for Respiratory Research, Department of Medicine, University College Medical School, Rayne Institute, London, United Kingdom; 2 Department of Epidemiology, Harvard School of Public Health, Boston, Massachusetts, United States of America; 3 Spanish Pneumococcal Reference Laboratory, Centro Nacional de Microbiologia, Instituto de Salud Carlos III, Majadahonda, Madrid, Spain; 4 Department of Anatomy, University College London, London, United Kingdom; 5 Department of Microbiology, Tumor and Cell Biology, Karolinska Institutet and the Swedish Institute for Infectious Disease Control, Stockholm, Sweden; 6 Department of Infectious Disease Epidemiology, St. Mary's Hospital Campus, Imperial College London, London, United Kingdom; National Institute of Allergy and Infectious Diseases, National Institutes of Health, United States of America

## Abstract

**Background:**

Immunity to infections caused by *Streptococcus pneumoniae* is dependent on complement. There are wide variations in sensitivity to complement between *S. pneumoniae* strains that could affect their ability to cause invasive infections. Although capsular serotype is one important factor causing differences in complement resistance between strains, there is also considerable other genetic variation between *S. pneumoniae* strains that may affect complement-mediated immunity. We have therefore investigated whether genetically distinct *S. pneumoniae* strains with the same capsular serotype vary in their sensitivity to complement mediated immunity.

**Methodology and Principal Findings:**

C3b/iC3b deposition and neutrophil association were measured using flow cytometry assays for *S. pneumoniae* strains with different genetic backgrounds for each of eight capsular serotypes. For some capsular serotypes there was marked variation in C3b/iC3b deposition between different strains that was independent of capsule thickness and correlated closely to susceptibility to neutrophil association. C3b/iC3b deposition results also correlated weakly with the degree of IgG binding to each strain. However, the binding of C1q (the first component of the classical pathway) correlated more closely with C3b/iC3b deposition, and large differences remained in complement sensitivity between strains with the same capsular serotype in sera in which IgG had been cleaved with IdeS.

**Conclusions:**

These data demonstrate that bacterial factors independent of the capsule and recognition by IgG have strong effects on the susceptibility of *S. pneumoniae* to complement, and could therefore potentially account for some of the **differences** in virulence between strains.

## Introduction

The nasopharyngeal commensal *Streptococcus pneumoniae* commonly causes severe infections such as pneumonia, meningitis and septicaemia. Immunity to *S. pneumoniae* is highly dependent on the complement system [1], [2], [3], [4], a series of host serum and cell surface proteins organised into three enzyme cascades termed the classical, alternative and mannan binding lectin (MBL) pathways. The classical pathway is activated by specific antibody [5], and by recognition of *S. pneumoniae* cell wall phosphorylcholine (PC) by natural IgM or the serum pentraxin proteins C reactive protein (CRP) and serum amyloid P (SAP), or by binding of the capsule to the lectin SIGN-R1 [2], [6], [7], [8]. Classical pathway activation results in binding of C1q to the bacterial surface and the formation of the classical pathway C3 convertase [5]. MBL binds poorly to *S. pneumoniae* and may have little effect on complement activation by *S. pneumoniae* [2], [9]. The alternative pathway is spontaneously activated unless the target cell is coated in sialic acid or complement inhibitory proteins such as factor H (FH) [5]. Complement activation leads to C3b deposition on the bacterial surface which is further processed to iC3b, both of which act as opsonins for phagocytosis [5]. Complement activation also aids the inflammatory response through release of anaphylaxins such as C5a and improves adaptive immune response to *S. pneumoniae* through direct stimulation of B cells by C3d. As a consequence neutrophil phagocytosis and killing of *S. pneumoniae* and optimum antibody responses are highly dependent on complement activity [10], [11].

The importance of complement for immunity to *S. pneumoniae* is further demonstrated by the multiple mechanisms of complement evasion that *S. pneumoniae* has evolved. The extracellular polysaccharide capsule of *S. pneumoniae* inhibits classical pathway and alternative pathway activity and inhibits degradation of C3b to iC3b [12]. Various *S. pneumoniae* proteins also inhibit complement activity, including the choline binding surface proteins PspA and PspC, the toxin pneumolysin, pneumococcal histidine triad proteins (Pht), and the exoglycosidases NanA, BgaA, and StrH [13], [14], [15], [16]. PspA inhibits both alternative and classical activity by unknown mechanisms, whereas PspC prevents alternative pathway activity by binding the host alternative pathway regulator protein Factor H (FH) and in some strains the classical pathway inhibitor C4b binding protein (C4BP) [13], [14], [17], [18], [19]. Extracellular release of pneumolysin may divert classical pathway activity away from *S. pneumoniae* by binding C1q. Inhibition of complement activity by Pht proteins depends on serotype background and could be related to FH binding [13], [16], [20]. How exoglycosidases affect complement activity is not clear but could be due to deglycosylation of complement protein glycoconjugates [15]. *S. pneumoniae* can also degrade C3 [21]) and there are probably other *S. pneumoniae* mechanisms of complement evasion that have yet to be described.

Different *S. pneumoniae* strains vary in their sensitivity to complement [22], [23]. There are over 90 recognized capsular serotypes related to the type, order and chemical bonds of monosaccharide units within the polysaccharide chain and the presence of side chains. Using capsular switched strains expressing different capsular serotypes on the same genetic background, we and others have demonstrated that variations between *S. pneumoniae* strains in complement sensitivity is at least partially linked to serotype [24], [25], [26]. In addition, during infection *S. pneumoniae* can undergo phase variation between transparent variants which have relatively thin capsules and are more sensitive to complement and opaque phase variants which have thicker capsules and are more complement-resistant [26]. Conversely, antibody recognition of the capsule could increase complement activity via activation of the classical pathway, and the sensitivity of different *S. pneumoniae* capsular serotypes to anti-capsular antibody varies. For example serotype 19F requires a greater concentration of anti-capsular antibody to achieve similar levels of killing in an opsonophagocytosis assays as serotypes 6B or 23F [23].

As well as the capsule there is also a surprising amount of other genetic variation between *S. pneumoniae* strains. Only around 60% of gene clusters are common to all strains and gene content varies up to 10% between any two strains, even between strains with the same capsular serotype [27], [28]. This genetic variation could influence bacterial complement resistance independent of capsular serotype. For example the expression of complement-inhibiting proteins may vary between strains, and there is significant allelic variation in PspA and PspC structure between strains that may have functional consequences [14], [29], [30]. Furthermore, *S. pneumoniae* genetic variation between strains includes numerous gene deletions and insertions, with at least 41 regions of diversity (RD) containing clusters of genes present only in restricted strains [28], [31], [32], [33] some of which could influence complement activity. Recent publications have confirmed that non-capsular genetic variation influences *S. pneumoniae* complement resistance. C3b/iC3b deposition has marked variation between different serotype 6A strains [34], and Melin et al. showed differences in complement sensitivity between three different genetic backgrounds expressing capsular serotypes 19F or 6B [23]. The central role of complement for immunity to *S. pneumoniae* suggests these differences in complement sensitivity could be clinically relevant, and indeed complement resistant strains have been linked with a higher potential to cause otitis media or death during invasive infection [34], [35].

To confirm and further characterise the effects of genetic variation independent of capsular serotype on complement-mediated immunity we have used several clinical *S. pneumoniae* isolates with different genetic backgrounds assessed by multilocus sequence typing (MLST) [36] for eight common capsular serotypes. Using these strains we have investigated the relationship between antibody binding, neutrophil association, capsule thickness and *S. pneumoniae* resistance to opsonisation with C3b/iC3b.

## Results

### C3b/iC3b deposition varies between S. pneumoniae strains with the same capsular serotype

**Figure 1:**
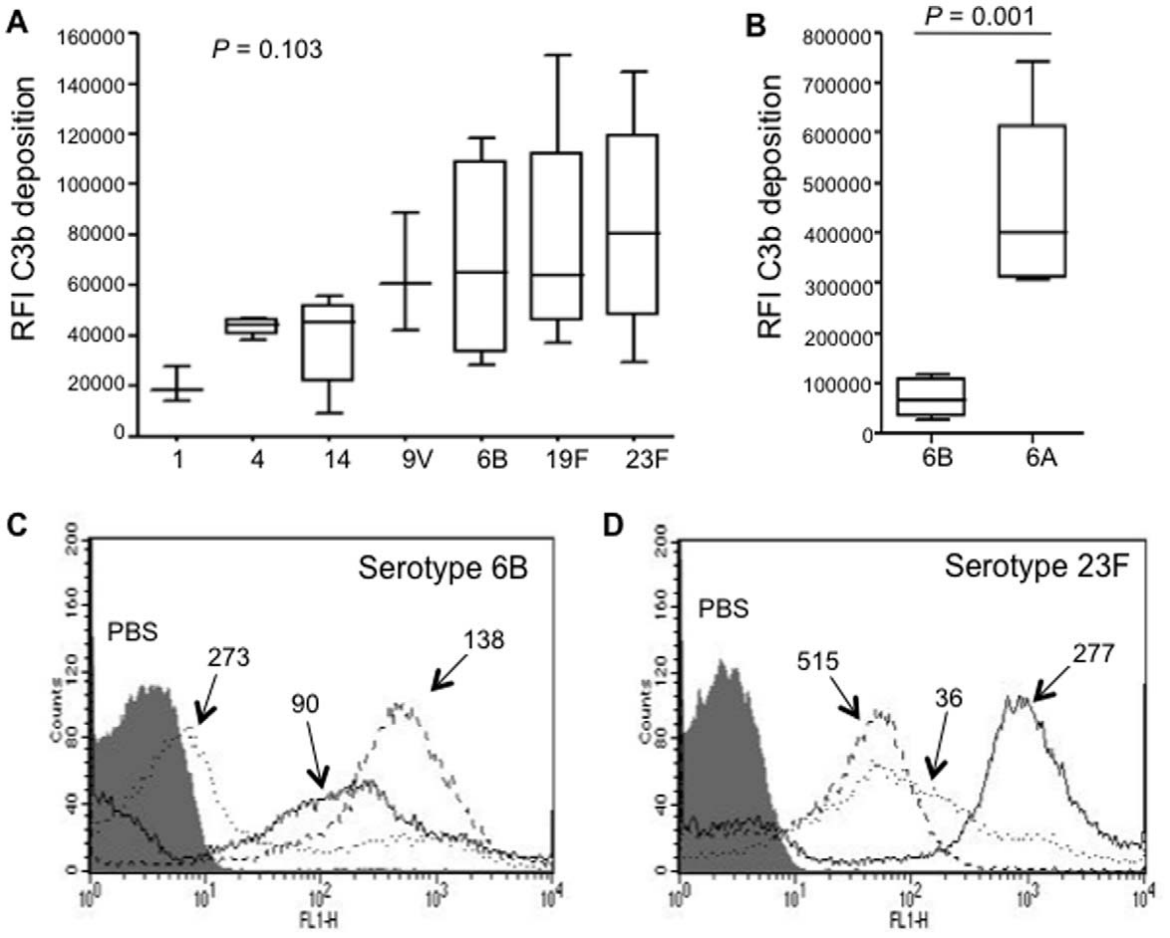
C3b/iC3b deposition in 25% human serum on clinical isolates of *S. pneumoniae* measured using flow cytometry and expressed as an FI. (*A*) Median (IQR) FI of C3b/iC3b deposition on at least 3 clinical *S. pneumoniae* isolates from serotypes 1, 4, 7F, 9V, 6B, 19F and 23F. Data were analysed using the Kruskal Wallis test and showed no significant differences between serotypes shown in this panel. (*B*) Median FI of C3b/iC3b deposition on clinical isolate *S. pneumoniae* strains of serotype 6B and 6A. *P* value represents the results of a Mann-Whitney U Test. (*C*) and (*D*) Representative flow cytometry histograms of C3b/iC3b deposition on serotype 6B and 23F strains. Labels refer to strain ST.

To investigate the relative importance of genetic variation independent of capsular serotype between *S. pneumoniae* strains on complement activity, C3b/iC3b deposition was assessed on 33 clinical isolates from eight different capsular serotypes that frequently cause *S. pneumoniae* infections (1, 4, 14, 6A, 6B, 9V, 19F and 23F). For each serotype, three or more strains representative of dominant sequence types (STs) identified by MLST were chosen, with a total of 24 different STs investigated. Five pairs of strains with the same capsular serotype and same ST but isolated from distinct geographical areas were also included (Table 1). There was considerable variation in the results of C3b/iC3b deposition between the strains. When the results of C3b/iC3b deposition were presented as a composite fluorescence index (FI) to represent both the proportion of bacteria coated with C3b/iC3b and the intensity of C3b/iC3b deposited, the results varied from 9060 (for an ST162 serotype 14 strain) to 743240 (for an ST1068 serotype 6A strain) (Table 1). For some serotypes, strains with different STs had markedly different results (Table 1 and Fig. 1). For example, the serotype 6B ST138 strain had markedly higher levels of C3b/iC3b deposition than the serotype 6B ST90, and ST273 strains (Fig. 1C, ANOVA with post hoc tests *Ρ*<0.001), and the serotype 23F ST277 strain had greater C3b/iC3b deposition than the serotype 23F ST515 and ST36 strains (Fig. 1D, ANOVA with post hoc tests *Ρ*<0.001). Other serotypes showed less variation in C3b/iC3b deposition between strains (eg serotypes 1 and 4) (Fig. 1). There were also differences in C3b/iC3b deposition for strains with the same sequence type (ST) and with the same capsular serotype that were isolated from different geographical backgrounds (eg ST176 serotype 6B isolates, *P*<0.001 Student's T test) (Table 1). There were no significant differences in median C3b/iC3b deposition for each serotype except for serotype 6A strains which had a higher median C3b/iC3b deposition than all other serotypes (comparison to 6B strains shown in Fig. 1B). These data demonstrate that *S. pneumoniae* strain background independent of capsular serotype can be a strong determinant of C3b/iC3b deposition, causing such large variations in results between strains with the same capsular serotype that differences in median C3b/iC3b deposition between capsular serotypes are obscured.

**Table 1:** Clinical isolates of *S. pneumoniae* used in this study with their ST, and the results for C3b/iC3b deposition (FI in 25% serum), IgG binding (FI 25% human serum), C1q binding (FI 25% human serum), and neutrophil association (percentage association in 20% human serum).

Serotype	ST	Original strain name	IgGBinding(FI ± SD)^a^	C1q Binding(FI ± SD)^a^	C3b/iC3bBinding(FI ± SD)^a^	% neutrophil association(± SD)	source^b^
1	306	BHN 30	1150±250	4360±130	27600±5930	63.9±4.5	BHN
1	228	BHN 32	1060±50	1470±120	14000±3260	59.8±3.1	BHN
1	217	BHN 166	1090±40	2790±265	18340±4000	46.2±2.8	BHN
4	1222	BHN 42	1360±30	1530±370	45590±1780	55.5±3.1	BHN
4	205	BHN 43	1650±50	3850±520	38170±1720	48.7±3.2	BHN
4	205	n/a^c^	1440±80	3380±625	47130±9080	68.7±4.1	BGS
4	259	n/a	1390±40	1820±490	43060±13090	56.4±3.9	BGS
6A	488	IOKOR1277-3	4470±560	6700±390	484860±10920	65.0±0.9	BGS
6A	490	IOKOR1373-9	1800±340	3740±330	313230±9730	75.7±2.2	BGS
6A	518	IOKOR801-2	2000±460	4690±390	307290±8370	47.1±4.2	BGS
6A	1068	IO13048	6940±80	7480±170	743240±13850	94.6±0.6	BGS
6B	90	M225-6B	2920±740	2590±40	38460±8910	66.7±4.2	BGS
6B	138	M7-6B	1700±330	5990±430	83550±10240	65.0±5.0	BGS
6B	138	BHN 49	4000±200	6780±160	118330±1820	95.9±1.9	BHN
6B	176	M49-6B	2100±20	3690±330	46780±6980	62.0±3.2	BGS
6B	176	BHN 50	1870±90	5480±70	99540±6760	88.3±2.4	BHN
6B	273	JJ270-6B	1160±300	1720±60	28330±3500	57.4±0.9	BGS
9V	156	BHN 62	1260±90	3380±100	88814±10140	66.9±4.2	BHN
9V	162	BHN 63	1040±100	4340±130	41809±8320	76.3±3.0	BHN
9V	162	BHN 69	1250±290	4710±880	60744±7590	78.3±2.5	BHN
14	124	M117-14	1550±30	4280±140	48640±3840	71.1±5.4	BHN
14	124	BHN 84	1520±180	1960±110	45224±4790	52.4±4.2	BGS
14	162	M65-14	1660±180	790±140	9060±1490	45.0±0.2	BGS
14	156	M134-14	2700±220	2720±300	55800±8190	59.2±0.3	BGS
14	307	PJ581/14	2800±260	4300±170	34510±6850	75.4±1.4	BGS
19F	162	BHN 100	1170±120	4220±980	36980±4000	63.3±1.9	BHN
19F	236	BHN 388	1810±130	4220±600	73880±11990	80.3±2.8	BHN
19F	425	BHN 97	800±10	4480±690	54470±3950	63.7±2.2	BHN
19F	556	n/a	2610±260	6350±700	151280±800	93.5±5.6	BHN
23F	36	OXC-1417-23F	1450±130	3300±550	67080±8280	55.2±4.1	BGS
23F	37	IOPR1592	2810±220	4030±240	94130±9770	71.9±1.9	BGS
23F	277	JJ279-23	3450±170	6350±430	144780±8240	92.3±5.3	BGS
23F	515	IOKOR706-5	650±30	910±60	29250±1190	45.0±0.5	BGS

a FIs are expressed as arbitrary units.

b BHN, Birgitta Henriques-Normark; BGS, Brian G. Spratt.

c not available.

### Neutrophil association correlates with the results for C3b/iC3b deposition

**Figure 2:**
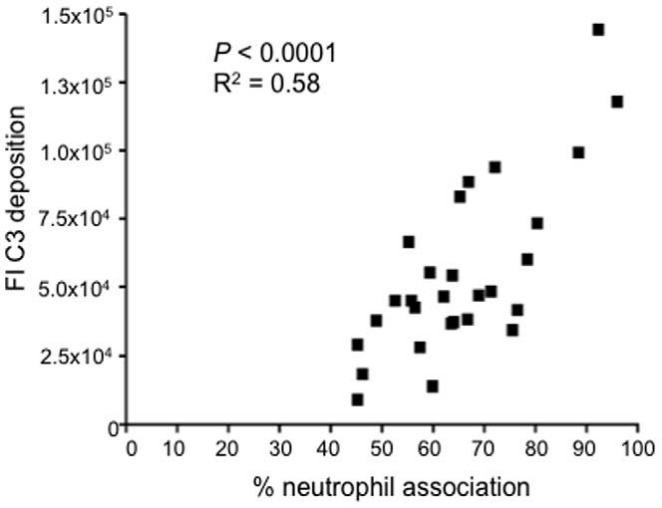
Correlation of neutrophil association in 20% human serum measured using flow cytometry of clinical isolates of *S. pneumoniae* with the FI for C3b/iC3b deposition. Neutrophil association results are expressed as percentage of neutrophils associated with bacteria. Serotype 6A strains were excluded from this correlation due to their very high level of C3b/iC3b deposition results and their higher level of IgG binding (see Figure 4). *P* values and R^2^ were obtained using Pearson's correlation test.

To assess whether the differences in C3b/iC3b deposition between *S. pneumoniae* strains had functional consequences, neutrophil association after incubation in human serum was measured using a flow cytometry assay. Results were expressed as the proportion of neutrophils associated with fluorescent bacteria which has previously been shown to be mainly due to phagocytosis [10], [12]. Neutrophil association varied significantly between *S. pneumoniae* strains, with a minimum of 45% of neutrophils associated with a ST515 serotype 23F strain and a maximum of 96% of neutrophils associated with a ST138 serotype 6B strain in the test conditions used (Table 1). Like the C3b/iC3b deposition results, there were large variations in neutrophil association results between strains with the same capsular serotype and there were large overlaps for the results of individual strains from different serotypes (Table 1). Importantly, C3b/iC3b deposition on each *S. pneumoniae* strain had a positive correlation with the neutrophil association results (Pearson's correlation co-efficient R^2^ = 0.58) (Fig. 2), demonstrating that variation in C3b/iC3b deposition between strains was functionally important for neutrophil mediated immunity.

### Differences in C3b/iC3b deposition between strains of the same serotype are unrelated to capsule thickness

**Figure 3:**
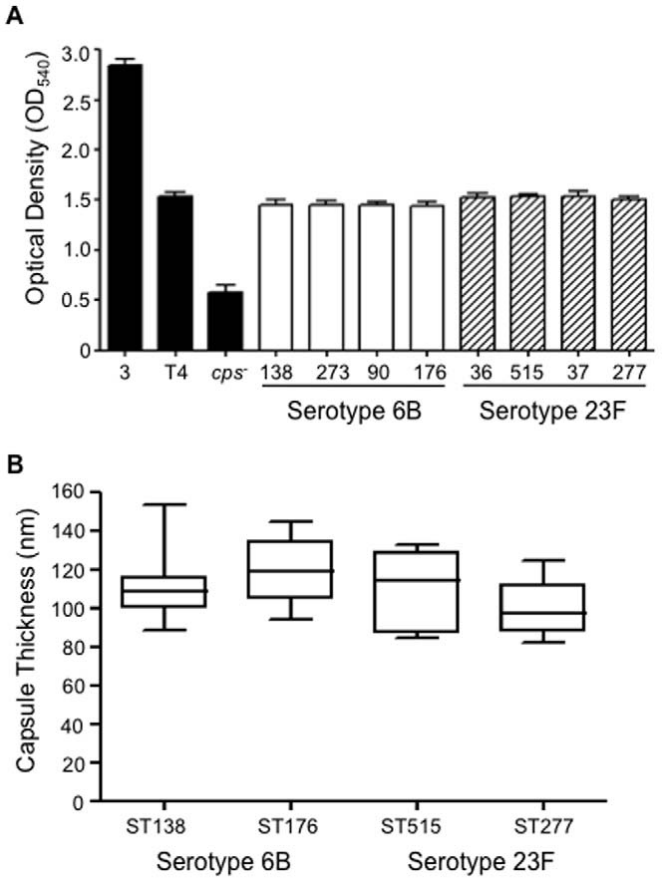
Capsule thickness of selected *S. pneumoniae* clinical isolates. (*A*) OD_540_ for the All-Stains assay for serotype 6B and 23F *S. pneumoniae* strains compared to results for the TIGR4 serotype 4 strain (T4), the 0100993 serotype 3 strain (3), and an unencapsulated TIGR4 strain (*cps^−^*). There were no statistically significant differences between strains within each serotype. (*B*) Medians and interquartile range (IQR) of capsule thickness (nm) measured by EM for *S. pneumoniae* serotypes 6B (ST 138 and 176) and 23F (ST 515 and 277). There were no statistically significant differences between strains within each serotype.

*S. pneumoniae* undergoes phase variation between opaque and transparent variants, with the latter having thinner capsule layers [37] and higher levels of C3b/iC3b deposition [26]. Phase variation therefore could potentially explain some of the differences in C3b/iC3b deposition between strains with the same capsular serotype. However, microscopic examination on transparent medium found the all strains used for this study were in opaque phase. To further investigate whether variation in capsule thickness may explain differences in C3b/iC3b deposition between strains, the capsule quantity of the 6B or 23F serotype clinical isolates (which had marked variation in C3b/iC3b results between strains) was assessed using the All-Stains assay. For both serotypes there were no significant differences between strains in the amount of polysaccharide detected. These data were supported by EM measurement of the capsule layer thickness for selected strains (the technique is too labour intensive to be used for large numbers of strains) using a fixation technique that preserves the capsule. Previously we have demonstrated that EM can identify significant differences in capsular width between between opaque and transparent phase variants of the same capsular serotype [26]. EM confirmed that two serotype 6B and two serotype 23F strains with large differences in C3b/iC3b deposition had no significant differences in capsule thickness (*Ρ*>0.05, Fig. 3B). Furthermore FITC dextran exclusion microscopy showed no differences in bacterial size for all the serotype 6A, 6B and 23F strains investigated in this study (data not shown). Hence differences in C3b/iC3b deposition between strains of the same capsular serotype are unrelated to major variations in capsule thickness.

### Correlation of antibody recognition and C3b/iC3b deposition results

**Figure 4:**
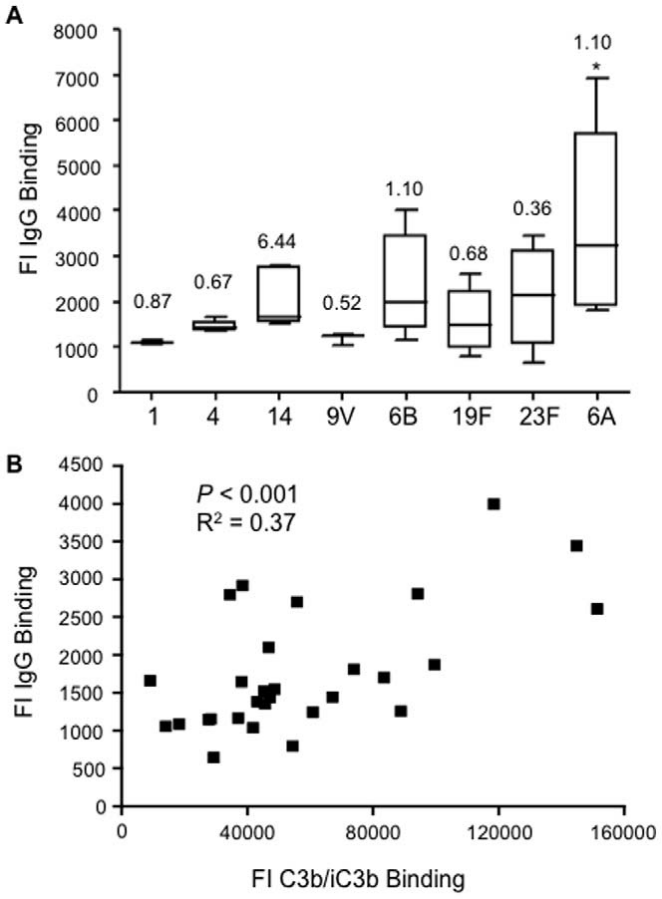
Capsular antibody levels in serum used for this study measured using ELISAs and total IgG binding in 25% serum on *S. pneumoniae* clinical isolates measured using flow cytometry. (*A*) Median (IQR) FI of IgG binding on at least 3 clinical *S. pneumoniae* isolates from serotypes 1, 4, 7F, 9V, 6B, 19F and 23F. For the overall comparison between strains *P*<0.034 (Kruskal Wallis) and compared to serotype 1 *P<0.05 for 6A strains only (Dunn's post-hoc tests). Total anti-capsular IgG levels (µg ml^−1^) are given above each box and whisker plot for each serotype. (*B*) Correlation of the FI for IgG binding to the FI for C3b/iC3b deposition on clinical isolates of *S. pneumoniae*. Serotype 6A strains were excluded from this correlation due to their very high level of C3b/iC3b deposition results (see Figure 1). *P* values and R^2^ were obtained using Pearson's correlation test.

Complement deposition on *S. pneumoniae* is highly dependent on antibody recognition [12], [23]. To assess whether differences in C3b/iC3b deposition between strains could be related to differences in recognition by naturally acquired antibody, anti-capsular antibody levels in the serum used for the complement assays were measured using ELISA. In addition, to measure combined anti-capsular and anti-protein antigen antibody recognition of each strain in the sera used for this study total IgG binding was assessed using a flow cytometry assay. Although there was some variation between serotypes, with the exception of serotype 14, anti-capsular IgG levels were less than 1.10 µg ml^−1^ for each serotype (Fig. 4A) in the serum used for the complement assays. Anti-serotype 14 levels were markedly higher at 6.44 µg ml^−1^; however there was no obvious relationship between anti-capsular antibody levels and C3b/iC3b deposition. For example serotypes 6B, 19F and 23F had very similar median C3b/iC3b deposition results despite different levels of anti-capsular IgG (Figs. 1 and 4A). There was also significant variation in total IgG binding to strains within the same serotype (eg 14, 6A, 6B, 19F, and 23F) when incubated in the serum used for the complement assays; these serotypes tended to have large variations in C3b/iC3b deposition between strains as well. Median IgG binding to the serotype 6A strains was significantly higher than median IgG binding to other capsular serotypes, perhaps partially explaining why the serotype 6A strains were particularly sensitive to complement (Fig. 4A). Overall, total IgG binding to each strain showed a relatively weak positive correlation with C3b/iC3b deposition that was strongly statistically significant (Pearson's Correlation Co-efficient R^2^ = 0.37, *P*<0.001) (Fig. 4B).

### Relationships between IgG and C1q binding and C3b/iC3b deposition results for each strain

**Figure 5:**
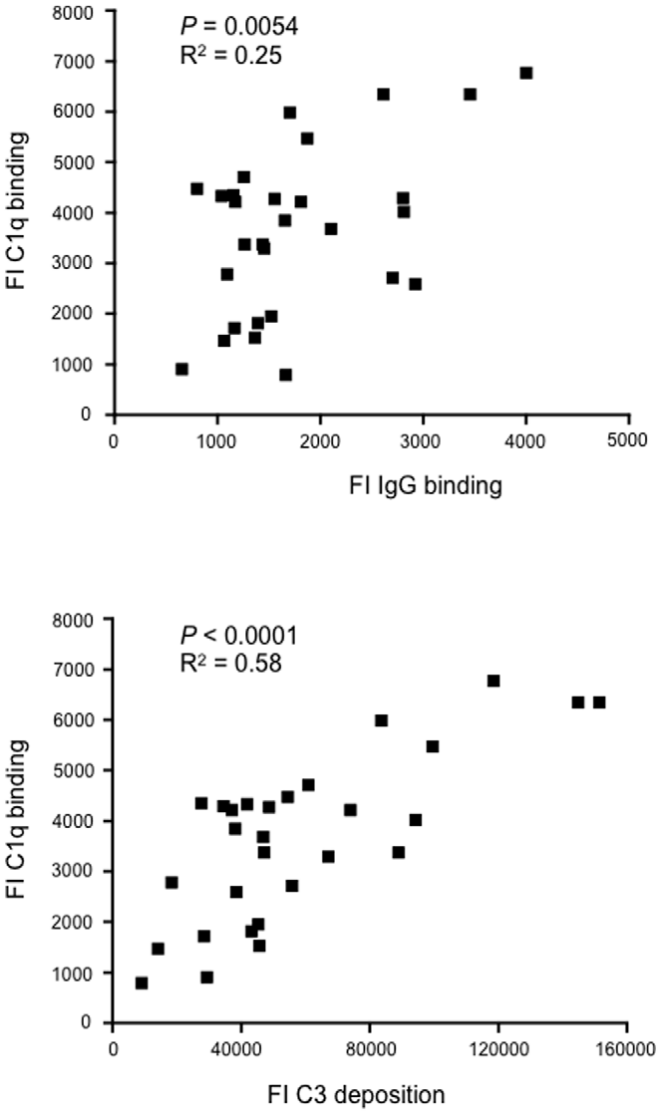
Correlations of C1q binding to *S. pneumoniae* clinical isolates in 25% human serum measured using flow cytometry. Correlations of C1q binding to *S. pneumoniae* clinical isolates in 25% human serum measured using flow cytometry to IgG binding (A) and C3b/iC3b deposition results (B). Serotype 6A strains were excluded from these correlations due to their very high level of C3b/iC3b deposition results and their higher level of IgG binding (see Figure 4). *P* values and R^2^ were obtained using Pearson's correlation test.

C1q is the first component of the classical complement pathway and binds to *S. pneumoniae* through antibody recognition as part of the adaptive immune response or binding of serum proteins such as CRP and SAP to the cell wall as part of the innate immune response. Binding of C1q to *S. pneumoniae* varied markedly between strains (Table 1 and Fig. 5), and correlated with total IgG binding. However the correlation between C1q and total IgG binding was weak (R^2^ of 0.25, *P*>0.001), whereas the correlation between C1q binding and C3b/iC3b deposition was relatively strong (R^2^ 0.58) (Fig. 5). These data suggest that total IgG binding was not the only determinant of differences in C3b/iC3b deposition between *S. pneumoniae* strains and that other factors affecting C1q binding may also be involved.

### Differences in C3b/iC3b deposition between strains of the same serotype persist in the absence of IgG

**Figure 6:**
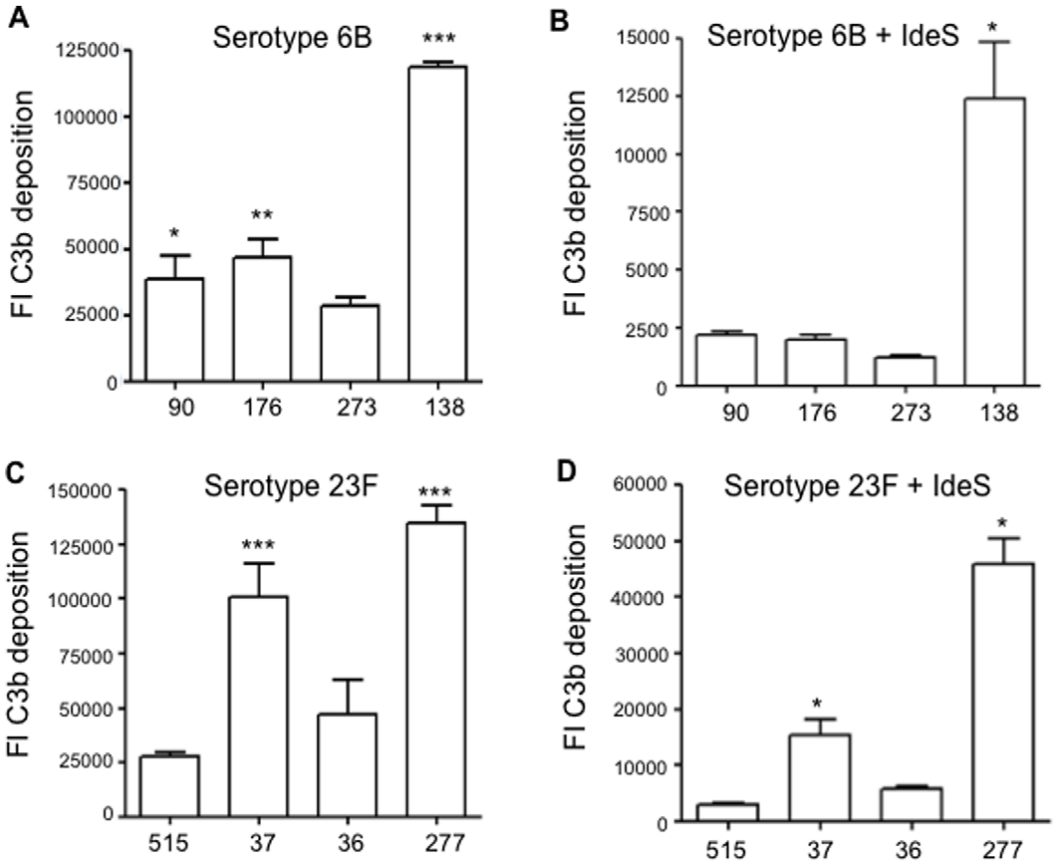
C3b/iC3b deposition on *S. pneumoniae* independent of IgG. (*A*), (*C*) Mean FI of C3b/iC3b deposition measured using flow cytometry on (*A*) 6B and (*C*) 23F clinical isolates of *S. pneumoniae* when incubated in untreated 25% human serum. (*B*), (*D*) Mean FI of C3b/iC3b deposition measured using flow cytometry on (*B*) 6B and (*D*) 23F clinical isolates of *S. pneumoniae* when incubated in 25% human serum treated with IdeS. For all panels, error bars represent SDs and *P<0.01 and **P<0.001 for results compared to the ST273 (A and B) and 515 (C and D) strains (ANOVAs with post-hoc tests).

To further investigate whether differences in IgG binding are not the only cause of variations in C3b/iC3b deposition between strains of the same capsular serotype, the C3b/iC3b assays were repeated for the serotype 23F and 6B strains using serum in which the IgG had been cleaved by IdeS. IdeS totally abrogates IgG binding to *S. pneumoniae*, so should remove any differences in complement activation due to variable antibody recognition of each strain [12], [38]. As expected C3b/iC3b deposition was lower on all strains in IdeS treated serum, and some of the differences between strains were also reduced (eg between serotype 23F ST37 and ST515 or ST36 strains) (Fig. 6). However significant differences between some strains with the same serotype persisted (eg between serotype 6B ST138 and the serotype 23F ST277 strains and other strains with the same serotype, ANOVAs with post-hoc tests *Ρ*<0.01) (Fig. 6B and 6D), confirming that at least some of the variation in C3b/iC3b results between strains of the same serotype is not due to differences in IgG binding.

## Discussion

The vital role of complement for immunity to *S. pneumoniae* [1], [2], [3], [4], [10], [39] suggests that differences in complement sensitivity could affect the relative invasiveness of *S. pneumoniae* strains, and so evaluating factors influencing *S. pneumoniae* complement resistance is an important area of research. Data obtained using capsular serotype switched strains has confirmed capsular serotype is one factor that affects the sensitivity of different *S. pneumoniae* strains to complement [24], [25], [26]. Recent data has demonstrated that there is also a considerable amount of genetic variation between strains within a serotype and even within strains with the same MLST genotype [27], [28], [31], [32], [33]. We have therefore investigated the relationship of capsule serotype and non-capsular genetic variation to complement resistance using a range of strains with representative STs for eight of the commonest serotypes that cause invasive disease [40]. The results of the C3b/iC3b deposition assays demonstrated that complement resistance varied markedly between strains for some capsular serotypes (eg 6A, 6B, 19F, 23F), with less variation between strains for other serotypes (eg 1 and 4). The functional importance of these differences in C3b/iC3b results was demonstrated by their strong positive correlation with neutrophil association, one of the main effectors for bacterial killing during *S. pneumoniae* infection.

Our data support other recently published data demonstrating that variations in sensitivity to complement-mediated immunity between different *S. pneumoniae* strains is affected by both capsular serotype and genetic variation independent of capsular serotype [23], [24], [26], [34], [35]. For example, Sabharwal et al. found that C3b/iC3b deposition varied between serotype 6A strains [34] and Melin et al. very recently demonstrated C3b/iC3b deposition varied between strains with the same capsular serotypes [35]. Our data support these findings but in general show larger variations between strains for some serotypes. This may be partially due to the slightly larger number of isolates analysed by Melin et al (6 or 7 for some serotypes), but is also likely to be partly caused by presentation of our results as an FI rather than just using the geometric mean MFI of C3b/iC3b deposition. FI is the product of the proportion of bacteria positive for C3b/iC3b and the mean intensity MFI, allowing both factors to be represented which is important for data with a biphasic distribution (Fig. 1) [2] but increases the range of results obtained. The large differences in C3b/iC3b deposition results for strains with the same serotype meant the only significant difference in median C3b/iC3b deposition between serotypes was between serotype 6A strains and all other serotypes. Hence our data suggest that for clinical strains serotype-independent factors are often just as powerful influences on C3b/iC3b deposition as capsular serotype. As well as differences in C3b/iC3b deposition between strains with different STs, there were also differences for strains with the same ST and capsular serotype. Therefore obtaining an accurate median level of C3b/iC3b deposition for each serotype using data obtained from a relatively small number of representative strains is not really possible, as using serotype and ST alone may not adequately identify a range of representative strains. Instead comparing phenotypes such as complement sensitivity between each capsular serotype will require investigating a very large number of clinical strains for each serotype or using capsular switched strains [24], [26], [41].

Several mechanisms may explain variation in C3b/iC3b deposition independent of capsular serotype. The most obvious would be differences in capsule thickness or antibody activity between strains, and we have investigated these possibilities for selected strains showing large differences in C3b/iC3b deposition. We found no differences in capsule thickness between serotype 6B and 23F strains with large variations in sensitivity to complement, and antibody to capsular polysaccharide should have identical effects for strains with the same capsular serotype. Total IgG binding, which includes binding to protein and other non-capsular antigens that may vary in expression between strains of the same capsular serotype, did weakly correlate with C3b/iC3b deposition. However, large differences in C3b/iC3b deposition between some strains with the same serotype persisted when IgG was depleted by cleavage with IdeS [26], [38], indicating that there must be additional mechanisms affecting C3b/iC3b deposition. Serotypes with large variations between strains in the C3b/iC3b deposition results tended to be those that have previously been described as more genetically diverse (eg serotypes 6A, 6B, 19F), whereas those with less variation in C3b/iC3b results were more clonally related (1 and 4) [42]. These data are compatible with a genetic basis for the differences in complement sensitivity, such as allelic variation of *pspC* and *pspA* affecting the corresponding proteins' functions [29], [30] or differences in expression levels of genes encoding PspC, PspA, pneumolysin and other proteins that affect complement activity. For example, only some PspC variants bind to C4BP, which could cause differences in sensitivity to classical pathway (C1q dependent) immunity [18]. In addition, Factor H binding to PspC varies between strains and we have recently demonstrated that deletion of *pspC* has different effects on C3b/iC3b deposition depending on strain background [14], [43]. The large number of RDs in the *S. pneumoniae* genome may contain genes that also directly affect complement function in specific strains only, and the surface expression of complement targets (which are largely unknown for *S. pneumoniae*) could also vary between strains. Finally there could be as yet not described small differences in capsule structure within a serotype or additional unrecognised phase variant phenotypes which could affect complement activity. The close correlation between C1q binding and C3b/iC3b deposition is compatible with differences in complement resistance between strains being related to differences in classical pathway activation. However, the mechanisms affecting serotype-independent differences in complement resistance between *S. pneumoniae* strains are likely to be complex and vary between strains, making their characterisation difficult. Investigating some of the seemingly closely related strains (same ST and serotype) with large differences in C3b/iC3b deposition identified by this study might begin to characterise these mechanisms.

Given the vital role for complement in preventing systemic infection by *S. pneumoniae* [2], [13], differences in C3b/iC3b deposition could account for some of the recognised differences in invasiveness between *S. pneumoniae* strains. Relative invasiveness is closely linked to capsular serotype, and serotype-dependent differences in complement sensitivity have been correlated to serotype-dependent variations in death rates [35]. Linking capsule-independent genetic variation to specific infection phenotypes is difficult due to the low proportion of total isolates that belong to each ST. However, there are some STs that seem to be more invasive than other STs with the same capsular serotype, and it is possible that this could be related to serotype-independent differences in complement sensitivity [42]. For example, transparent phase 6A strains isolated from the middle ear in patients with otitis media were more resistant to complement than transparent phase 6A strains isolated from the nasopharynx [34]. Although isolation of a particular strain from the nasopharynx does not mean it is necessarily a non-invasive isolate, these data are compatible with the possibility that infection develops with particular strains due to their complement resistant phenotype. Similar data obtained for large numbers of well-characterised strains for other common serotypes may also help to assess any links between invasiveness and complement resistance.

To conclude, in this manuscript we have demonstrated that the genetic background of *S. pneumoniae* strains caused marked variations in opsonisation with C3b/iC3b independent of capsular thickness or serotype. This capsule-independent variation in complement resistance was similar in strength to capsular serotype-dependent effects. Variation in complement resistance was partially dependent on differences in IgG binding, but persisted for some strains even in IgG-depleted serum and was strongly correlated to C1q binding. These data indicate capsule-independent genetic variation between strains affects interactions with complement. Further investigation is required to characterise the mechanisms causing variation in complement sensitivity between *S. pneumoniae* strains and its relevance during the development of disease.

## Materials and Methods

### Ethics

Human serum was obtained with written consent from healthy human volunteers under ethical approval granted by the local University College London ethics committee. As serum donors were university staff, written consent was considered unnecessary.

### Bacterial strains and culture conditions

*S. pneumoniae* strains used in these experiments from are shown in Table 1. All strains were clinical isolates obtained from nasopharyngeal culture of children or from invasive infection in adults and children and were previously serotyped and assigned an ST by MLST as described [42], [44], [45]. Bacteria were cultured at 37°C in 5% CO_2_ on blood agar plates or in Todd-Hewitt broth supplemented with 0.5% yeast extract to an optical density at 580 nm of 0.4 (approximately 10^8^ CFU/ml) and stored at −70°C in 10% glycerol as single-use aliquots. Bacterial phase was determined using transparent medium (Tryptone Soya with catalase) under magnification and oblique, transmitted illumination as previously described [46]. Capsule quantity was determined using the All-Stains assay for acidic polysaccharides. Bacteria grown to an optical density at 580 nm of 0.4 were washed and resuspended in water and chloroform added. After shaking the aqueous layer was added to All-Stains (Sigma) solution (10 mg in 50 mls of formamide and 30 µl acetic acid) and the optical density (OD_640_) measured [46].

### Complement and neutrophil association assays

Pooled human serum was obtained from unvaccinated normal human volunteers [12], [26]. Sera were stored in single use aliquots at −70°C. To remove active IgG, sera were treated as previously described with 1% Immunoglobulin G-degrading enzyme (IdeS, a kind gift from Drs Mattias Collin and Lars Björck, Lund University) which cleaves IgG at the hinge region [38], for 45 minutes at 37°C before use. Serum levels of capsule-specific antibody titres were measured using standardized ELISAs (http://www.vaccine.uab.edu/ELISA%20Protocol.pdf). Briefly, serum was mixed with an absorbent containing C-polysaccharide (C-PS) and 22F capsular PS to neutralize antibody binding to C-PS and other common contaminants before addition in serial dilutions to ELISA plates previously absorbed with individual capsular serotype antigens. Serotype specific antibody bound to the ELISA plate was detected with anti-human IgG antibody conjugated with alkaline phosphatase, followed by addition of the substrate, *p*-nitrophenyl phosphate and reading the optical density at 405 nm. Serum total IgG binding to *S. pneumoniae* using flow cytometry and a R-phycoerythrin goat anti-human IgG (Jackson ImmunoResearch) as described [10]. C3b/iC3b deposition and C1q binding to *S. pneumoniae* were measured using previously described flow cytometry assays and fluorescein isothiocyanate (FITC) conjugated polyclonal anti-human C3 or anti-C1q (ICN) [12], [13], [26], [39]. Results of complement and IgG binding assays are presented as a fluorescence index (FI, percentage of positive bacteria multiplied by the geometric mean MFI of IgG or C3b/iC3b binding) in arbitrary units [2], [10]. To ensure consistent results assays were repeated using two or more stock sources cultured at different times for each strain. Neutrophil association was investigated using an established flow cytometry assay, fresh human neutrophils (10^5^ per reaction) and *S. pneumoniae* (10^6^ per reaction) labelled with 6-carboxyfluorescein succinimidyl ester (FAMSE; Molecular Probes) and incubated in serum (diluted in PBS) for 20 min at 37°C [10], [47]. A minimum of 10,000 cells analyzed using flow cytometry to identify the proportion of neutrophils associated with bacteria [39].

### Electron Microscopy and FITC-Dextran exclusion measurement of capsule width

For EM analysis of the capsule width bacteria were processed as described by Hammerschmidt et al [48]. Briefly, mid-log phase *S. pneumoniae* were incubated at 37°C for 20 mins in serum or PBS then fixed in 1% PFA. PFA-treated bacteria were fixed twice with 2% formaldehyde and 2.5% glutaraldehyde in cacodylate buffer containing 0.075% ruthenium red (plus 0.075 M lysine-acetate for the first fixation only), and then with 1% osmium in ruthenium red containing cacodylate buffer for 1 h at room temperature. Washed samples were then dehydrated with a graded series of ethanol, infiltrated with LRWhite acrylic resin then pure resin before the blocks were baked in gelatin capsules, cut into ultrathin sections and mounted on copper or nickel grids. Sections were counterstained with 1% aqueous uranyl acetate and 0.19 M lead citrate before air drying and examination with a Zeiss EM 1010 transmission electron microscope (100 kV). Image J software used to determine capsule thickness by measuring the cross-sectional area of 10 or more randomly chosen bacteria including and excluding the capsule, and the areas used to calculate the capsule layer width (assuming circularity) [12]. FITC-Dextran exclusion measurement of bacterial size was performed as described [49], with diameters measured for 10+ bacteria for all of the serotype 6A, 6B and 23F strains.

### Statistics

C3b/iC3b deposition assays were repeated using different strain stocks by separate laboratory workers who were unaware of any previous results. Results for each strain were highly reproducible. Flow cytometry data between individual strains were analysed using unpaired Student's T test (comparison of two samples) or one way ANOVAs (comparisons of multiple samples) with post-hoc tests and presented as means (SD). EM and pooled flow cytometry data from multiple strains were compared using the Kruskal Wallis test with Dunn's multiple comparison test (multiple groups) and presented as medians (IQRs). Correlations were performed using Pearson's correlation test.
